# Effects of Shape, Roughness and Gloss on the Perceived Reflectance of Colored Surfaces

**DOI:** 10.3389/fpsyg.2020.00485

**Published:** 2020-03-20

**Authors:** Vanessa Honson, Quan Huynh-Thu, Matthew Arnison, David Monaghan, Zoey J. Isherwood, Juno Kim

**Affiliations:** ^1^Sensory Processes Research Laboratory, School of Optometry and Vision Science, University of New South Wales, Sydney, NSW, Australia; ^2^Canon Information Systems Research Australia (CiSRA), Sydney, NSW, Australia; ^3^Nearmap, Sydney, NSW, Australia; ^4^Bandicoot Imaging Sciences, Sydney, NSW, Australia; ^5^School of Psychology, University of Wollongong, Wollongong, NSW, Australia

**Keywords:** material appearance, surface properties, gloss, lightness, color, 3D shape, virtual reality

## Abstract

This study examined perceptual differentiation of specular from diffuse shading for the recovery of surface color and gloss. In Experiment 1, we parametrically varied the mesoscale relief height of globally planar surfaces, specular sharpness and the orientation of the surface relative to the light source. We obtained psychophysical matches for perceived color saturation and value (HSV), but also considered whether the main effects could be influenced by color space used when transforming data to perceptually-uniform CIE LCH space. Results revealed strong interactions between perceived color attributes and the lighting conditions, the structure of specular reflections, and surface relief. Declines in saturation were observed with increasing specular roughness (using an HSV color representation), but no similar decline was observed in chroma (using a CIE LCH color representation). Experiment 2 found strong negative correlations between perceived gloss and specular roughness. Perceived gloss also depended on mesoscopic relief height and orientation of the surface relative to the light source. Declines in perceived gloss moderately accounted for the variability in color saturation and value matches obtained in Experiment 1. We found information about perceived specular coverage could further improve the model’s accountability of perceived color saturation and lightness (Experiment 3). These findings together suggest that perceived color saturation and color value depends on the visual system’s ability to distinguish the underlying diffuse shading from specular highlights in images.

## Introduction

Surfaces vary in shape, color, gloss and a host of other properties (e.g., texture and opacity). Representing color and lightness attributes of surfaces on a computer monitor is a challenging problem because perceptual interactions are known to exist with shape (Schmid and Anderson, 2014) and gloss (Xiao and Brainard, 2008). In this study, we examined how perceived color saturation and lightness varies as a function of a surface’s relief height, orientation and glossiness. We also examine how perceived color attributes can vary differentially based on the type of color model used to represent the perceived color of surfaces simulated in graphical displays.

Graphical simulation of surfaces with material properties requires images to be rendered based on the optics underlying the reflectance of surfaces in the real world. The formation of natural images depends on complex interactions between the structure of prevailing illumination, 3D surface shape and reflectance, as well as the viewing direction. Much of this structure in images can be modeled using an idealized bi-directional reflectance distribution function (BRDF) (Nicodemus, 1965). Separate diffuse and specular components within this model respectively characterize different reflectance properties of surfaces. The diffuse component is determined by Lambertian reflectance, which is viewpoint-*independent* shading generated by the orientation of surface normals relative to the light source. Diffuse shading contains information tied to surface shape and color/albedo. The specular component describes the viewpoint-*dependent* shading generated by the orientation of surface normals relative to both the viewing direction and the prevailing light source(s). Specular shading is not only informative of surface gloss, but also the 3D shape of surfaces (Fleming et al., 2003, 2004).

**FIGURE 1:**
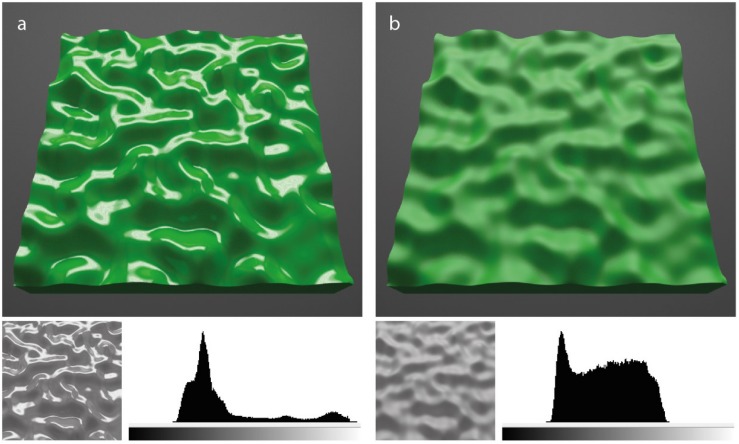
Renderings of a green tile showing different levels of 3D shape (megascale, mesoscale, and microscale). Megascale shape refers to the global form of the tile, a planar rectangular prism. Mesoscale relief refers to the visible perturbations in surface bumpiness. Microscale relief refers to the fine roughness that is visible when varying specular roughness between images **a** and **b** (Beckmann microfaceted specular roughness parameters of 0.05 and 0.40, respectively). The lower insets show the grayscale for luminance levels for the same central surface patch in the two images. Luminance histograms reveal large differences in the variability of photometric distributions between the surface patches.

Due to the dependence of both diffuse and specular shading on 3D shape, it is helpful to define the general spatial scales over which shape can be described. Previous authors have described surface shape at three main spatial scales: megascale, mesoscale, and microscale (Ho et al., 2007). Figure 1 depicts renderings of a surface with these three different levels of surface geometry. Megascopic shape refers to the overall global shape of the 3D object, which in this case is a rectangular prism or globally planar tile. The other terms refer to different aspects of surface relief. Mesoscopic shape refers to the visible surface geometry and can be thought of as the irregularity of visible surface texture, most often referred as ‘bumpiness’ (Ho et al., 2007, 2008; Marlow et al., 2012; Qi et al., 2015). The decline in clarity of the specular highlights between Figures 1a,b is due to microscale roughness of the surface. For convenience, this microscale relief is commonly simulated using the specular lobe (or specular roughness) of the BRDF, rather than the diffuse roughness (e.g., Mooney and Anderson, 2014).

An observer relies on specular and diffuse components to recover information about the gloss and lightness (or color) of surfaces, but the ability of their visual system to tap into the information carried by these different image layers depends on their perceptual separability (Barrow and Tenenbaum, 1978). This task of attributing image structure to physical causes in the environment is complicated by evidence that both diffuse and specular components differentially contribute to the perception of potentially multiple surface properties. For example, the perception of surface shape is well known to depend on the structure of diffuse shading (i.e., shape from shading), but also depends on the structure of specular reflections (e.g., Fleming et al., 2003; Mooney and Anderson, 2014; Kim and Marlow, 2016). Also, the perception of gloss can depend on the geometric relationship between specular orientation and adjacent patterns of diffuse shading (Beck and Prazdny, 1981; Todd et al., 2004; Kim et al., 2011, 2012; Marlow et al., 2011). Hence, the perception of surface and material properties is a highly complex problem of inverse optics (see Anderson and Kim, 2009; Fleming, 2014). Both diffuse and specular components can contribute differentially to the perception of gloss and lightness/color, but are conflated in images. Understanding how we separate these sources of image structure perceptually remains a challenge for vision science.

Some studies have attempted to simplify the problem of explaining material appearance by using image statistics to understand how the visual system recovers information about surface gloss and lightness (Motoyoshi et al., 2007; Sharan et al., 2008). Motoyoshi et al. (2007) proposed that surface gloss and lightness depend on underlying computations of image statistics; perceived gloss increases (and lightness decreases) when increasing image skew whilst holding mean luminance constant. For example, based on this proposal, the surfaces depicted in Figure 1 appear to vary in perceived lightness because their underlying luminance histograms differ from one another; the surface with sharp specular highlights appears darker because its luminance histogram is more positively skewed, whereas the surface with rougher specular highlights appears lighter because its luminance histogram is less (positively) skewed. Sharan et al. (2008) obtained similar findings by instructing observers to judge the lightness of real surfaces with relief depicted in photographs. Lightness judgments were obtained using a physical Munsell scale. They found that perceived lightness judgments were more veridical when surfaces were more complex by containing significant mesostructure and specularity. They also proposed that the errors in perceived lightness could be explained by a linear combination of different statistical parameters of filtered images, including skew.

Rather than depending on image statistics, other evidence has shown that the perception of gloss and lightness depends on the *structure* of luminance variations in images. Previous studies have shown that specular edges are correlated with diffuse shading, which depends on the alignment of their edges relative to isophotes – lines of isoluminance in diffuse shading (e.g., Koenderink and van Doorn, 1980). Breaking this “orientation” congruence has been shown to not just decrease perceived gloss, but caused the specular reflections to appear as changes in diffuse reflectance and the surfaces as pigmented (Beck and Prazdny, 1981; Todd et al., 2004; Anderson and Kim, 2009; Kim et al., 2011, 2012, 2014; Marlow et al., 2011). These findings support the possible interpretation that residual image structure not correctly attributed to specular reflectance can influence the perception of a surface’s lightness or color.

In addition to the orientation of specular reflections relative to shading, perceived gloss also depends on the sharpness of these reflections. Decreasing specular edge sharpness is known to decrease perceived gloss (Hunter and Harold, 1987; Pellacini et al., 2000; Fleming et al., 2003; Wendt et al., 2010; Kim et al., 2012). Kim et al. (2012) found that blurring specular reflections did not just reduce perceived gloss, but also that the blurred reflections “appeared less like specular reflections and more like diffuse shading” (p. 1593). In a recent study, Kim et al. (2016) showed that adapting observers to specular contours generated subsequent declines in perceived gloss, which they attributed to a dependence on neurally encoding information about sharp specular contours. These findings together suggest that accurate sensory coding of specular edge sharpness is necessary for both the detection and accurate classification of specular reflections. The accuracy of this perceptual classification is not only critical for the perception of gloss, but also the perception of lightness and color.

There is good evidence to suggest that perceived gloss is highly influenced by the complex ways that specular image structure can be constrained by surface relief. Marlow et al. (2012) showed that the perception of gloss in planar simulated surfaces with relief depends on complex interactions between relief height, surface reflectance and illumination. They showed that perceived gloss was non-linearly related to changes in the amplitude of mesoscopic surface shape, but these perceived changes could be explained by weighted linear combinations of observer judgments of specular sharpness, contrast and coverage within the image (see also Marlow and Anderson, 2013). Baar et al. (2016) used physical surface samples to examine the relationship between gloss perception and mesoscopic surface shape. Similar to some studies using rendered images (e.g., Ho et al., 2008), they found that surfaces with greater mesoscopic shape (but equated sheen) were perceived as glossier. However, unlike previous studies, their findings did not agree to the reverse; perception of 3D relief was found to be unaffected by increases in the sheen of the surfaces they used in their study.

Other studies have shown that the perception of lightness tends to be more veridical when the complexity of surfaces increases in mesoscopic shape and specular reflectance (Sharan et al., 2008; Schmid and Anderson, 2014). The surfaces used in these studies generated specular reflections that were locally sharp, consistent with surfaces that lack microscopic variations in shape. Xiao and Brainard (2008) found that the perception of color was somewhat invariant when transforming a globally convex spherical surfaces from matte to glossy. Observers generally estimated color independently of the photometric changes in image intensity caused by the addition of specular reflections. However, when they increased specular roughness to simulate microscale relief, the perceived gloss declined and perceived lightness increased. It is possible that the reciprocal effects of specular roughness on perceived gloss and lightness observed by Xiao and Brainard (2008) depended on differences in the perceptual performance of separating diffuse and specular components from one another. Any residual unclassified specular content could ultimately be conflated with the diffuse shading component, on which judgments of lightness/color are ultimately based. However, Xiao and Brainard (2008) did not examine these effects across changes in mesoscopic relief height, a scale at which specular image structure is known to strongly depend on shape (e.g., Ho et al., 2007; Marlow et al., 2012).

The theoretical motivation for this project is that the perceptual separability of specular and diffuse image content depends on the sharpness of specular reflections. Previous research has shown that participants base their judgments of lightness on brighter diffusely shaded surface regions (Toscani et al., 2013; Toscani and Valsecchi, 2019). When surfaces generate specular reflections, participants tend to ignore brighter surface regions covered by specular highlights when making judgments of a surface’s body color and lightness (Kim et al., 2012; Toscani et al., 2017). However, it is possible that they will tend to base their judgments on brighter image regions containing specular highlight when their edges are blurred, which is known to make specular highlight boundaries difficult to distinguish (Kim et al., 2016). We tested whether increasing the specular roughness of surfaces with mesoscopic relief can cause specular content to be mis-attributed to Lambertian reflectance, thus influencing perceived color saturation and value (Experiment 1). We verified whether any effects can be explained by observed changes in perceived gloss (Experiment 2) and changes imposed on perceived specular coverage (Experiment 3).

## Experiment 1

Previous research observed biases in perceived lightness when increasing specular roughness of globally convex spherical objects (Xiao and Brainard, 2008). The surfaces used in that study were devoid of any mesoscopic variations in surface shape. Variations in specular sharpness were entirely attributed to microscopic shape cues. However, increase in mesoscopic relief height can increase the perceived sharpness of specular reflections when microscopic roughness is preserved (Marlow et al., 2012). It remains unclear how these mesoscopic and microscopic shape cues might differentially influence perceived color attributes of saturation and lightness.

In Experiment 1, we sought to ascertain how perceived color saturation and value might co-vary with changes in specular roughness and mesoscopic surface shape. We parametrically varied specular roughness and the amplitude of local variations in mesoscopic surface shape. If biases in perceived color depend on the sharpness of specular reflections, then increasing specular blur should generate biases in perceived saturation and lightness. Increasing mesoscopic relief height (and therefore curvature) will tend to increase specular sharpness, which should reduce the size of any potential effect of specular roughness on perceived color value.

### Materials and Methods

#### Observers

Twenty-five healthy adults participated in the study; all were aged over 18 years (age range 18 to 50) and had normal or corrected-to-normal vision. All participants were naïve to the aims of the study, except for three who were authors (QH-T, MA, and DM). All procedures adhered to the ethical principles outlined in the Declaration of Helsinki.

#### Stimuli

The upper face of a cube was initially subdivided into a 203 × 203 vertex mesh. The remaining four vertices that formed the other five faces of the cube were moved toward the upper face to simulate a square 3D tile 10 cm × 10 cm × 3cm (width × height × thickness). Mesoscale shape perturbations were introduced into the upper face by displacing each vertex along the orthogonal z-axis according to the values of a base cloud noise procedural texture in Blender 3D (Size: 1.0, Nabla: 0.03, and Depth: 1). The values of the texture displacement map were scaled by different amounts to vary the amplitude of undulations in mesoscopic surface shape along the z-axis. Subsequent smoothing was performed using the Corrective Smooth modifier in Blender (Factor: 1.0 and Repeat: 10). This smoothing improved the quality of the 3D modeling following displacement mapping.

**FIGURE 2:**
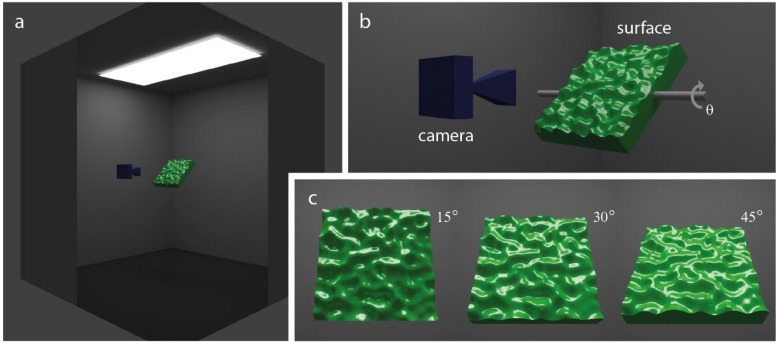
The simulated lighting environment. **(a)** The lighting environment was an enclosed viewing chamber fitted with an overhead rectangular area light. Note that the near corner of the viewing chamber has been cut away for the purposes of showing the arrangement of overhead lighting, the planar 3D surface (green), viewing camera (blue), and internal matte walls (Ref = 0.4) and floor (Ref = 0.1). **(b)** Surfaces were slanted obliquely, relative to the camera’s viewing direction, by angular rotations (θ) around the horizontal axis (shown in gray). **(c)** Sample images showing views of the surface slanted away from frontoparallel to the viewing direction by angles of 15°, 30°, or 45°.

The stimulated color of the 3D tile surfaces was always the same and set to a green hue in HSV color space (hue = 120°, saturation = 100%, value = 80%). Green was used as it is consistent with previous research on material perception (e.g., Ho et al., 2008). Surfaces were centered within a simulated lighting environment that was consistent with viewing chambers used in real-world psychophysical experiments on material appearance. Figure 2 provides an overview of the setup for simulated viewing and lighting conditions used in this experiment. The room was a cube (3.28 m^3^) with completely matte walls and floor. We used a large overhead rectangular emitter (2.5 m × 1 m) containing an additional two rectangular area lights 6 cm × 120 cm (Pure white each with Energy = 100) to generate natural primary lighting of surfaces embedded in our viewing chamber (Figure 2a). A camera with a focal length of 35 mm was situated 60 cm from the midpoint of the surface. This distance was appropriate to ensure the surfaces remained in full view across changes in its angular orientation of θ around the horizontal axis (Figure 2b). Figure 2c shows sample rendered images obtained for values θ of 15°, 30°, and 45°. This scene configuration generated images with little or no clipping of specular highlights within the color gamut, so no tone mapping was required.

**FIGURE 3:**
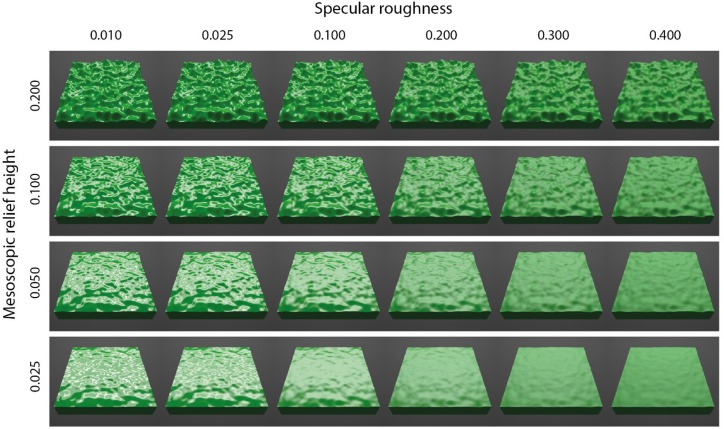
Rendered images of surfaces varying multi-parametrically in mesoscopic shape (across rows) and specular roughness (across columns). Images shown for the surface oriented at 45° in slant.

At each of the three surface orientations, we parametrically varied mesoscopic relief height and specular roughness as exemplified in Figure 3 for the 45°condition. We varied the mesoscopic relief height over four levels using the vertex displacement modifier in Blender (0.025, 0.050, 0.100, and 0.200). These values scaled the intensity range of the displacement map and generated undulations in mesoscopic shape with peak-to-peak amplitudes that approximately corresponded to 2.5%, 5%, 10%, and 20% of the surface’s width. The Corrective Smooth tool in Blender was used with 10 iterations to eliminate any artifacts in resulting surface geometry. We also parametrically varied specular roughness over six levels while holding specular amplitude constant (0.010, 0.025, 0.100, 0.200, 0.300, and 0.400). We used the Beckmann microfaceted distribution in the cycles render of Blender 3D to simulate specular roughness. This ensured that rough specular reflections tended to model the behavior more like diffuse reflectance rather than a mirror-like shiny surface with a narrow specular lobe (Guarnera et al., 2016). The range of specular roughness levels was chosen to be the same as used in previous research (Mooney and Anderson, 2014). Specular amplitude was held constant at 0.20, as used previously to generate the realistic glossy appearance of common natural materials (e.g., Marlow et al., 2012).

Images were rendered at the camera’s vantage point for each stimulus condition at a resolution of 2000 × 2000 pixels in 24-bit RGB bitmap format. The rendered images were generated in Cycles Render for Blender 2.77 using a python script to coordinate the rendering on a Dell Precision 5510 with Intel (R) Core i7-6820HQ CPU@2.70GHz computer. Path tracing was used with 128 render samples per pixel. The simulated light paths were set with default parameters for full global illumination. These rendering parameters were appropriate for generating images that could be sub-sampled to 800 × 800 using the Lanczos filter method in the custom stimulus presentation software for quality presentation with minimal noise on an Eizo CG275W monitor (27-inch diagonal with resolution 1920 × 1200 and 2.2 gamma). Images were rendered in sRGB color space within Blender 3D for presentation on this sRGB display. Images were viewed at a distance of approximately 70 cm for an effective size of approximately ± 10° visual angle (horizontal and vertical).

#### Procedure

Prior to participating, observers were informed that they would be required to make perceptual matches of surface color for planar surface images that were presented in a random order on a computer monitor. Training was offered for some observers to gain familiarity with what is required in a matching task. The pre-rendered images used in training were of a smooth planar surface devoid of mesoscopic surface changes presented on the left side of the display. Most of the observers were confident they understood the task after completing several trials. For the actual experiment, a total of 72 images were presented in a randomized order on the left side of the display (4 levels of relief height, 6 levels of specular roughness, and 3 levels of orientation relative to the light source).

**FIGURE 4:**
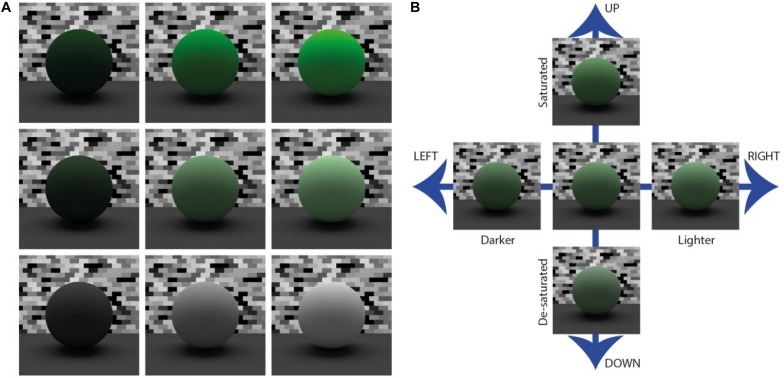
Spherical surfaces were used in the asymmetric color matching task. **A:** The surfaces were situated within the same light field as the bumpy planar surfaces, but a mural of random brick texturing was situated on the far wall behind the spheres. The sphere was also rendered on a flat plane that provided some ambient lighting to the underside of the sphere to increase the realism of the display. **B:** Selections were varied by pre-set steps in saturation (along columns) and value (along rows).

Perceptual matches of color saturation and value were made in HSV color space using pre-rendered images of a matte sphere devoid of specular reflections that was presented on the right side of the display (Figure 4). The sphere was seated on a tabletop plane (reflectance = 0.2) and a textured achromatic random brick pattern was tiled behind the sphere (reflectance range: 0.05 to 0.95). We used a sphere to ensure that the distribution of surface orientations was compatible with all three surface orientations of target planes. Observers used the arrow keys on a standard keyboard to move through a pre-rendered 11 × 11 matrix of images (11 levels of color value and 11 levels of color saturation). Horizontal keypresses increased or decreased color value (ranging 0.1 to 1.1). Vertical keypresses increased or decreased color saturation (ranging 0.0 to 1.0). The observer depressed the spacebar to record the setting that appeared to most closely match the color saturation and value of the target plane. Responses were recorded to ASCII file for subsequent data analysis.

#### Data Analysis

Observer settings of color saturation and value were separately averaged across observers for plotting purposes. The data were analyzed using a repeated-measures three-way Analysis of Variance (ANOVA) in the open-access statistical package R. This allowed us to test for any main effects of surface orientation, mesoscopic surface height or specular roughness on perceived color parameters of saturation and value.

**FIGURE 5:**
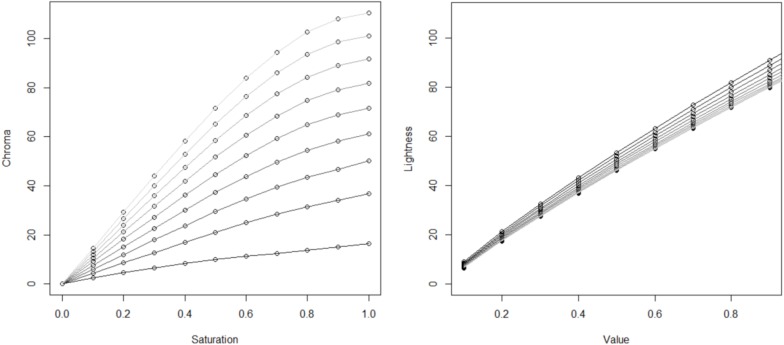
Transformation curves between HSV and CIE LCH space. The relationship between Chroma and Saturation for separate values in HSV **(left)**. The relationship between Lightness and Value for separate saturations in HSV space **(right)**. Note curves of increasing luminance correspond to increasing values and saturations.

One potential limitation is that the HSV color space may not be perceptually uniform for variations in saturation and value. Other representations of color such as CIE LCH space do maintain perceptual uniformity in representing observer judgments of color saturation and lightness. Figure 5 shows when the transformation for the color indices for a given Hue (120°) from HSV space (Saturation and Value) to CIE LCH space (Chroma and Lightness) is conducted, the transformation varies widely in linearity for Saturation/Chroma but is more linear for Lightness/Value. Hence, data obtained was re-analyzed by transforming the recorded settings in HSV color space to the perceptually orthogonal CIE LCH color space.

### Results and Discussion

**FIGURE 6:**
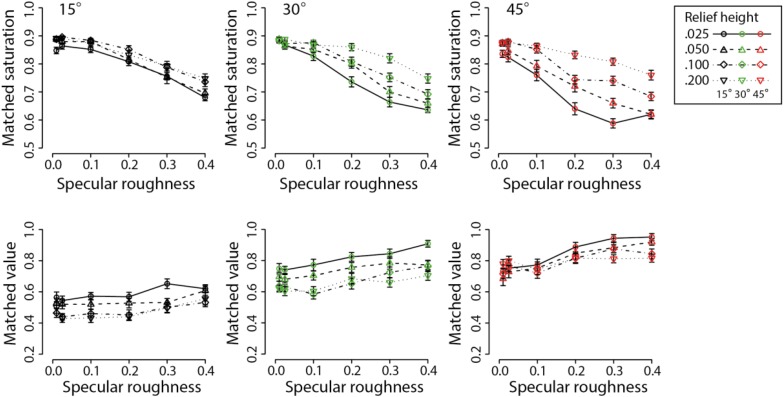
Means and standard errors showing results for color saturation matches **(upper row)** and color value matches **(lower row)**. Different line types and symbols shown in the legend correspond to data obtained at different relief heights for each of the three surface orientations: 15° (black points), 30° (green points), and 45° (red points).

The mean and standard errors for perceived chromatic saturation and value are plotted in Figure 6 against specular roughness for the three surface orientations. Separate curves show data for the different levels of relief height. Overall, the slopes suggest the emergence of a reciprocal relationship between color saturation and value with increasing specular roughness. The range of color saturation settings is seemingly greater for oblique as opposed to more frontal orientations.

For the color saturation data, a repeated-measures three-way ANOVA found significant main effects of surface orientation (*F*_2_,_48_ = 41.99, *p* < 0.00001), relief height (*F*_3_,_72_ = 128.2, *p* < 0.00001) and specular roughness (*F*_5_,_120_ = 272.3, *p* < 0.00001). There were also significant interactions between surface orientation and relief height (*F*_6_,_144_ = 20.61, *p* < 0.00001), surface orientation and specular roughness (*F*_10_,_240_ = 8.41, *p* < 0.00001), and relief height and specular roughness (*F*_15_,_360_ = 11.53, *p* < 0.00001). There was also a significant three-way interaction effect (*F*_30_,_720_ = 2.999, *p* < 0.00001).

For the color value data, a repeated-measures three-way ANOVA found significant main effects of surface orientation (*F*_2_,_48_ = 301.3, *p* < 0.00001), relief height (*F*_3_,_72_ = 39.82, *p* < 0.00001) and specular roughness (*F*_5_,_120_ = 36.49, *p* < 0.00001). There were also significant interactions between surface orientation and relief height (*F*_6_,_144_ = 6.43, *p* < 0.00001), surface orientation and specular roughness (*F*_10_,_240_ = 4.30, *p* < 0.00001), and relief height and specular roughness (*F*_15_,_360_ = 1.99, *p* < 0.05). There was no significant three-way interaction effect (*F*_30_,_720_ = 1.42, *p* = 0.069).

The declines in the perceived color saturation with increasing specular roughness are consistent with the findings of previous research using globally convex spherical surfaces (Xiao and Brainard, 2008). The additional effect of mesoscopic relief height on perceived color shows that the dependence of perceived color saturation and lightness on specular roughness is further influenced by the structure of mesoscopic surface relief. The interaction between relief height and surface orientation relative to the light source is consistent with the view that perceived surface color depends on complex interactions between illumination and surface optics. We propose this effect is due to a decline in the visual mis-attribution of specular content to diffuse shading.

**FIGURE 7:**
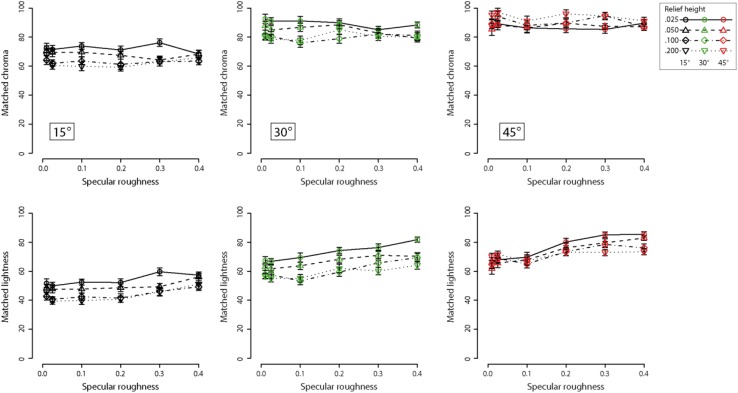
Means and standard errors showing chroma matches **(upper row)** and lightness matches **(lower row)** for increasing specular roughness. Different line types and symbols shown in the legend correspond to data obtained at different relief heights for each of the three surface orientations: 15° (black points), 30° (green points) and 45° (red points). Note these data are from Experiment 1 and are represented in CIE-LCH color space after transforming observer settings from HSV coordinates.

To assess whether the above relationships held under a more perceptually uniform color space such as CIE LCH, data was transformed for each of the observers scores from HSV to LCH by using a look-up table. The new scores were then averaged across participants and the same set of analyses were performed to examine main and interaction effects. The mean and standard errors for perceived chroma and lightness are plotted in Figure 7 against specular roughness for the three surface orientations. Separate curves show data for the different levels of relief height. In contradistinction to Figure 6, the slopes suggest a clear relationship between specular roughness and perceived lightness. No clear relationship between perceived chroma and specular roughness is apparent. However, there appears to be a relationship between viewing orientation and both lightness and chroma.

For the chroma matching task, a repeated-measures three-way ANOVA found significant main effects of surface orientation (*F*_2_,_48_ = 236.8, *p* < 0.00001), relief height (*F*_3_,_72_ = 9.79, *p* < 0.00005). However, there was no significant main effect of specular roughness on perceived chroma (*F*_5_,_120_ = 0.89, *p* = 0.49). There was a significant interaction between surface orientation and relief height (*F*_6_,_144_ = 18.3, *p* < 0.00001). However, there were no significant interaction effects between surface orientation and specular roughness (*F*_10_,_240_ = 1.54, *p* = 0.126), and relief height and specular roughness (*F*_15_,_360_ = 1.26, *p* = 0.23). There was no significant three-way interaction effect (*F*_30_,_720_ = 1.21, *p* = 0.20).

For the lightness matching task, a repeated-measures three-way ANOVA found significant main effects of surface orientation (*F*_2_,_48_ = 302.2, *p* < 0.00001), relief height (*F*_3_,_72_ = 41.58, *p* < 0.00001) and specular roughness on perceived lightness (*F*_5_,_120_ = 41.84, *p* < 0.00001). There was a significant interaction between surface orientation and relief height on perceived lightness (*F*_6_,_144_ = 6.13, *p* < 0.00001). There was also a significant interaction effect between surface orientation and specular roughness on perceived lightness (*F*_10_,_240_ = 4.42, *p* < 0.00005), and also a significant interaction effect between relief height and specular roughness on perceived lightness (*F*_15_,_360_ = 2.16, *p* < 0.01). There was no significant three-way interaction effect (*F*_30_,_720_ = 1.45, *p* = 0.06).

**FIGURE 8:**
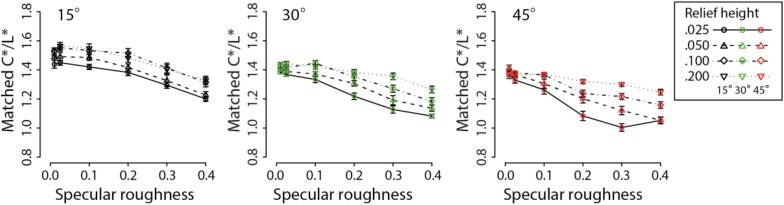
Means and standard errors showing matches on the basis of C^∗^/L^∗^ for increasing specular roughness. Different line types and symbols shown in the legend correspond to data obtained at different relief heights for each of the three surface orientations: 15° (black points), 30° (green points), and 45° (red points).

In contradistinction to the results with HSV, we found that increasing specular roughness had the effect of increasing perceived lightness and did not significantly influence perceived chroma after transforming the color representation to CIE LCH space. One potential reason for the absence of effect in perceived chroma when using CIE LCH space is that chroma is not synonymous with saturation in HSV space. According to Fairchild (2013), saturation refers to the estimated colorfulness of a surface patch proportional to its perceived brightness. Hence, saturation in CIE LCH space can be computed as C^∗^/L^∗^ (see Schiller and Gegenfurtner, 2016; Schiller et al., 2018). We therefore analyzed our transformed color matching data for C^∗^/L^∗^ in CIE LCH space to create a measure of similar to saturation in HSV color space. Figure 8 shows the transformed data for each of the three slant conditions.

For the C^∗^/L^∗^ transformation data, a repeated-measures three-way ANOVA found significant main effects of surface orientation (*F*_2_,_48_ = 149.9, *p* < 0.00001), relief height (*F*_3_,_72_ = 85.59, *p* < 0.00001) and specular roughness on perceived colorfulness of surfaces, i.e., C^∗^/L^∗^ (*F*_5_,_120_ = 188.3, *p* < 0.00001). There was no significant interaction between surface orientation and relief height on matched C^∗^/L^∗^ (*F*_6_,_144_ = 1.6, *p* = 0.15). However, there were significant interaction effects between surface orientation and specular roughness (*F*_10_,_240_ = 7.30, *p* < 0.00001) and between relief height and specular roughness (*F*_15_,_360_ = 9.03, *p* < 0.00001). There was also a significant three-way interaction effect (*F*_30_,_720_ = 2.75, *p* < 0.00001)

The findings of dependence in perceived colorfulness (i.e., chroma/lightness) and lightness on changes in mesoscopic surface relief height were in accordance with the HSV color space. The interaction effect between relief height and surface orientation relative to the light source was found consistently for all HSV and CIE LCH parameters (except for C^∗^/L^∗^). The consistency of this interaction effect supports the view that perceived surface color depends on complex interactions between illumination and surface optics. Again, we propose this interaction effect is due to a decline in the visual (mis)-attribution of specular content to shading generated by diffuse reflectance, an idea that we consider further in the next experiment based on our raw HSV matching data.

## Experiment 2

The previous experiment found that judgments of color saturation and value were highly dependent on specular roughness, mesoscopic relief and viewing conditions (surface orientation relative to the light source and observer). Perceived color saturation and value was most distorted when specular roughness was high and mesoscopic relief was low. Changes in relief height may have indirectly influenced the perceived specular roughness/sharpness. Indeed, perceived specular edge sharpness was found to be higher when relief height is larger (e.g., Marlow et al., 2012). Therefore, it is possible these illusions of color depended on the incomplete separation of specular reflections from diffuse shading when specular edges had increasing roughness. According to this view, some of the specular content may have been mis-attributed to the surface’s underlying diffuse reflectance, which gave rise to the experience of differences in surface color and lightness. If some of the specular energy were classified as diffuse shading in this way, then proportionally less classifiable specular content would be available for generating the experience of gloss. In Experiment 2, we test whether increasing specular roughness and reducing mesoscopic shape of our surfaces generates associated declines in perceived gloss.

### Materials and Methods

#### Observers

Eight observers participated in this experiment, all of whom had previously participated in Experiment 1 a few weeks earlier. Only two of these were authors (QH-T and MA). All procedures adhered to the ethical principles outlined in the Declaration of Helsinki.

#### Stimuli

We used planar surface images that were identical to those used in Experiment 1. However, because we used the paired-comparisons method here, we eliminated the 0.025 specular roughness level to reduce the number of trials. Images were presented side-by-side on the same display using a two-alternative forced-choice method. The images subtended the same visual angle as in the previous experiment. The same image conditions were used as in the previous experiment.

#### Procedure

We measured perceived surface gloss using the paired-comparisons method (e.g., see Kim et al., 2011, 2012). Observers were informed that they would need to select which of two images presented side-by-side on the computer monitor appeared glossier or shinier. Observers were instructed to use the LEFT/RIGHT arrow keys on the keyboard to indicate their preference on each trial. Their responses were recorded to ASCII file for subsequent analysis.

To minimize the number of trials in a session, we broke up the experiment into three sessions (one for each of three surface orientations): 15°, 30°, and 45° on separate times of the day. Hence, there were 380 counterbalanced trials for each surface orientation, based on the 5 levels of specular roughness and 4 levels of relief height (20 × 20 - 20). Image pairs were fully randomized, and we counterbalanced the order for performing blocks of trials at each surface orientation across observers. Although the paired images were presented for an unlimited period of time, observers tended to make their judgments within approximately 5 s. Observers took no longer than approximately 40 min to complete all three blocks of trials, which included the initial briefing and provision of instructions.

#### Data Analysis

We computed probability estimates of perceived gloss for each image in each condition by dividing the number of times the image was selected as glossier by the number of times it was presented on the display. Probability estimates of perceived gloss were analyzed by a series of repeated-measures two-way ANOVAs using the open-access statistical package R. We further determined whether there was any relationship between these estimates of perceived gloss and the observers’ judgments of perceived color saturation obtained in the previous experiment. This relationship was assessed using Pearson’s product-moment correlations.

Results are reported using the original HSV color space only. We focused on HSV because the matching task was configured to perform the task in this way as it was the simplest color space to use when instructing participants on making different dimensional settings for their color matches. We also focused on the raw HSV data as the main effects were identical between color spaces, but the interaction effects were more consistent using that color space in Experiment 1. The effect of specular roughness was also found to exert greater effects on perceived saturation (HSV space) compared with both chroma and C^∗^/L^∗^ (CIE LCH space).

#### Results and Discussion

**FIGURE 9:**
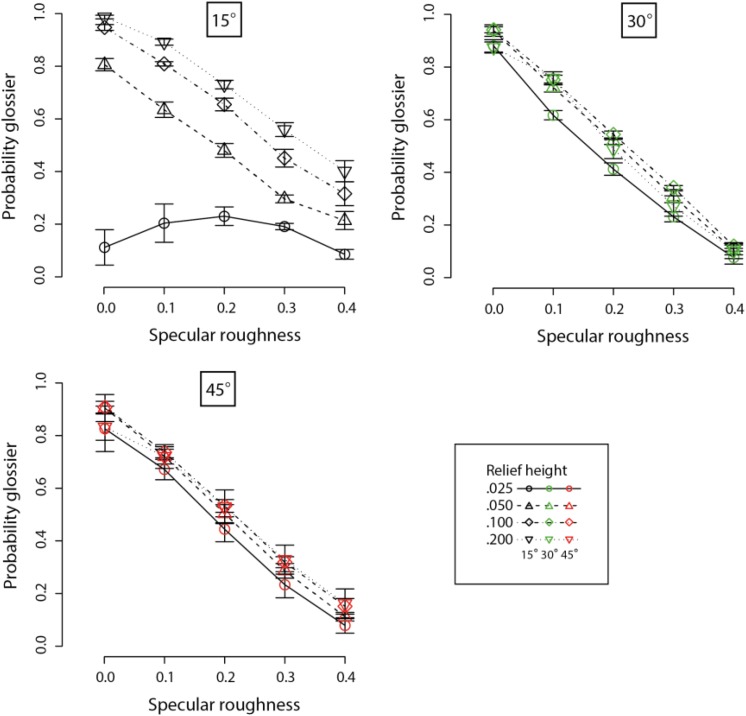
Means and standard errors of probability estimates for perceived gloss of planar surfaces varying in specular roughness and relief height. Different sets of axes are used to plot data for the three surface orientations relative to the light source from above (15° in black points, 30° in green points, and 45° in red points). Different line types and symbols shown in the legend correspond to data obtained at different relief heights.

The mean and standard errors for perceived gloss are plotted in Figure 9 against specular roughness for the three surface orientations. Separate curves show data for the different levels of relief height. We informally observe based on these plots that there are mostly consistent declines in perceived gloss with increasing specular roughness.

For the 15 surface orientation condition, a repeated-measures two-way ANOVA found a significant main effect of relief height on perceived gloss (*F*_3_,_21_ = 90.36, *p* < 0.00001). There was also a significant main effect of specular roughness on perceived gloss (*F*_4_,_28_ = 67.78, *p* < 0.00001). We further found a significant interaction effect on perceived gloss between relief height and specular roughness (*F*_12_,_84_ = 50.17, *p* < 0.00001).

For the 30° surface orientation condition, a repeated-measures two-way ANOVA did find a main effect of relief height on perceived gloss (*F*_3_,_21_ = 3.59, *p* < 0.05). There was also a significant main effect of specular roughness on perceived gloss (*F*_4_,_28_ = 1276, *p* < 0.00001). We further found a significant interaction effect on perceived gloss between relief height and specular roughness (*F*_12_,_84_ = 4.57, *p* < 0.00005).

For the 45° surface orientation condition, a repeated-measures two-way ANOVA found no significant main effect of relief height on perceived gloss (*F*_3_,_21_ = 0.70, *p* = 0.56). However, there was a significant main effect of specular roughness on perceived gloss (*F*_4_,_28_ = 219.6, *p* < 0.00001). There was no interaction effect between relief height and specular roughness on perceived gloss (*F*_12_,_84_ = 0.95, *p* = 0.50).

These results show there is a clear consistent decline in perceived gloss with increasing specular roughness, which is consistent with previous studies (e.g., Marlow et al., 2012). However, the effect of varying relief height on perceived gloss was less clear. Referring to Figure 9, there was a clear displacement between curves corresponding to data on different relief heights at 15°. This separation becomes less significant at higher surface orientations relative to the light source. Also note, the pattern of data is most dissimilar between the lowest relief height and the other levels of relief at 15°. Perceived gloss was lower and non-linear across changes in specular roughness when relief height was lower and when surfaces were oriented more frontally. This is evident in the significant interaction effect between specular roughness and relief height observed at smaller, but not larger, surface orientations.

Although increases in specular roughness reduced perceived gloss (Experiment 2) and reduced perceived color saturation (Experiment 1), the differences in perceived saturation across relief heights at more oblique surface orientations (e.g., 45 degrees) were not accompanied by similar differences in perceived gloss across changes in relief height at this surface orientation. It is possible this could be explained by failures in perceived roughness constancy across changes in viewing conditions (Ho et al., 2007). Nonetheless, we determined whether data on perceived color saturation and value from the previous experiment could be explained by gloss judgments obtained here in Experiment 2.

**FIGURE 10:**
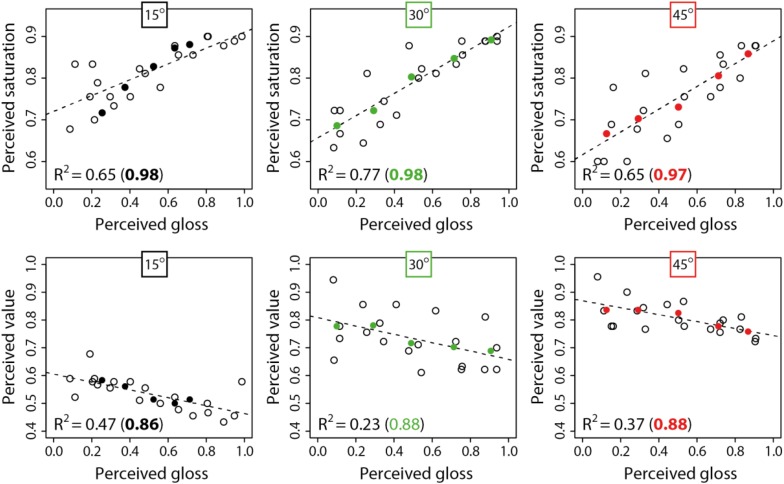
Mean perceived color saturation **(upper row)** and mean perceived color value. **(lower row)** plotted as a function of perceived gloss. Separate axes show data obtained for each of the three surface orientations relative to the light source (15°, 30°, and 45°). Hollow black circles show data points for all 20 conditions (relief height × specular roughness). Solid colored points show data averaged across relief height for the same level of specular roughness. R-squared values show the variability in color attributes accounted for by the pattern of variation in perceived gloss estimates (colored R-squared values after averaging across relief height). Dotted lines are linear least-squares fits to the data for all conditions.

Figure 10 plots the relationship between perceived color saturation as a function of perceived gloss for the same observers who participated in both Experiments 1 and 2. There were strong positive linear correlations between perceived color saturation and perceived gloss for the three surface orientations: 15° (*r* = 0.80, *t*_18_ = 5.75, *p* < 0.00005), 30° (*r* = 0.88, *t*_18_ = 7.79, *p* < 0.00001) and 45° (*r* = 0.81, *t*_18_ = 5.76, *p* < 0.00005). Comparatively weaker negative linear correlations were observed between perceived color value and perceived gloss for the three surface orientations: 15° (*r* = −0.69, *t*_18_ = 4.00, *p* < 0.001), 30° (*r* = −0.48, *t*_18_ = 2.30, *p* < 0.05) and 45° (*r* = 0.61, *t*_18_ = 3.25, *p* < 0.005).

The proportions of variability in perceived saturation and value accounted for by perceived gloss are shown in Figure 10 for data on all 20 conditions (hollow points). The reduced data obtained after averaging across relief heights at the same levels of specular roughness are also shown (solid points). The R-squared values were found to be consistently greater after averaging out the variability in relief height. The consistency of these differences suggests that perceived gloss accounts for a large proportion of variability in perceived color saturation and value that is imposed by variations in specular roughness. However, this gloss model accounted for a much smaller proportion of variability in these color attributes when relief height is allowed to co-vary.

Previously, Marlow et al. (2012) showed that perceived gloss could be predicted by the salience of image-based cues of specular contrast, sharpness and coverage. We directly manipulated sharpness in our experiments by parametrically varying specular roughness. However, it is possible that a subset of these image-based cues to gloss is relied upon differentially to segment and exclude specular highlights for the computation of color attributes (e.g., perceived coverage). In the next experiment, we consider whether information about perceived specular coverage might help to account for the variations in the perceived color saturation we observe.

## Experiment 3

Experiment 1 found that changing either specular roughness or surface relief height could have complex effects on perceived color saturation and value. In Experiment 2, we found that perceived gloss could account for much of the variability in perceived color saturation and value imposed by specular roughness alone, but not the variability introduced by changes in physical relief height. Such changes in relief height increase curvature, which will increase the range over which surface normals vary across a finite surface region. Increases in the range of surface normals will inevitably increase the number of surface regions with normals that bisect the angle formed between the viewing and illumination vectors, and therefore, the distribution of specular highlights across the surface. Hence, one potential image-based cue that could account for the pattern of data observed in Experiment 1, is the distribution of specular highlights across the surface (i.e., specular coverage). Surfaces with more frontal orientations and lower relief heights tend to have smaller regions of image space covered by specular reflections. Previous studies have found this coverage cue provides information that can differentially account for perceived gloss across a range of viewing conditions (Marlow et al., 2012). In Experiment 3, we obtained perceived specular coverage data on our own surface images to determine whether this image-based cue can help account for the variations in perceived color saturation generated by changes in both specular roughness and mesoscopic relief height.

### Materials and Methods

#### Observers

Five observers with normal or corrected-to-normal vision participated in this experiment. All but one observer were authors (VH, QH-T, MA, and DM). All procedures adhered to the ethical principles outlined in the Declaration of Helsinki.

#### Procedure

The procedure for the current experiment was identical to the previous paired-comparisons experiment, except for a change in instruction. Here, the task of the observers was to “select which of the two images appeared to have greater surface area covered by specular highlights.” Responses were recorded and analyzed using identical procedures as used in Experiment 2.

#### Results and Discussion

**FIGURE 11:**
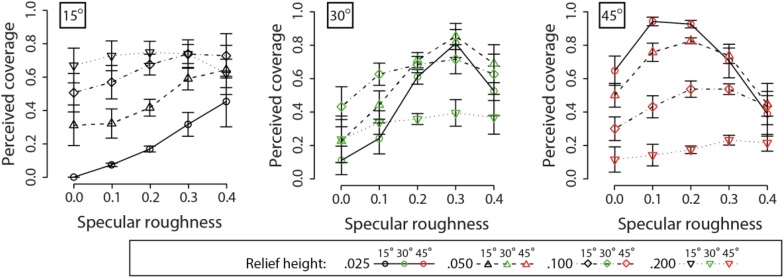
Means and standard errors for perceived coverage of surfaces by specular highlights. Data for different surface orientations relative to the primary lighting direction from above are plotted across separate axes. Different line types and points are used to plot data for different relief heights.

Figure 11 plots the perceived coverage estimates across changes in specular roughness and relief height for the three different surface orientations relative to the light source. Eyeballing these data, we can see there are complex interactions between surface orientation, relief height and specular roughness on perceived coverage.

For the 15° surface orientation condition, a repeated-measures two-way ANOVA found a significant main effect of relief height on perceived specular coverage (*F*_3_,_9_ = 22.76, *p* < 0.0005). However, there was no significant main effect of specular roughness on perceived coverage (*F*_4_,_12_ = 0.52, *p* = 0.72). There was a significant interaction effect on perceived coverage between relief height and specular roughness (*F*_12_,_36_ = 7.96, *p* < 0.00001).

For the 30° surface orientation condition, a repeated-measures two-way ANOVA found a significant main effect of relief height on perceived specular coverage (*F*_3_,_9_ = 31.03, *p* < 0.00005). However, there was no significant main effect of specular roughness on perceived coverage (*F*_4_,_12_ = 1.51, *p* = 0.26). There was a significant interaction effect on perceived coverage between relief height and specular roughness (*F*_12_,_36_ = 12.06, *p* < 0.00001).

For the 45° surface orientation condition, a repeated-measures two-way ANOVA found a significant main effect of relief height on perceived specular coverage (*F*_3_,_6_ = 28.8, *p* < 0.001). However, there was no significant main effect of specular roughness on perceived coverage (*F*_4_,_8_ = 1.60, *p* = 0.27). However, there was a significant interaction effect between relief height and specular roughness on perceived coverage (*F*_12_,_24_ = 11.6, *p* < 0.00001).

These data reveal there are very complex, though systematic, differences in perceived specular coverage across changes in relief height and surface orientation. When relief height was low, coverage was estimated to be progressively greater with increasing surface orientation away from the observer toward the light source. When relief height was high, coverage was estimated to be progressively lower with increasing surface orientation away from the observer toward the light source.

**FIGURE 12:**
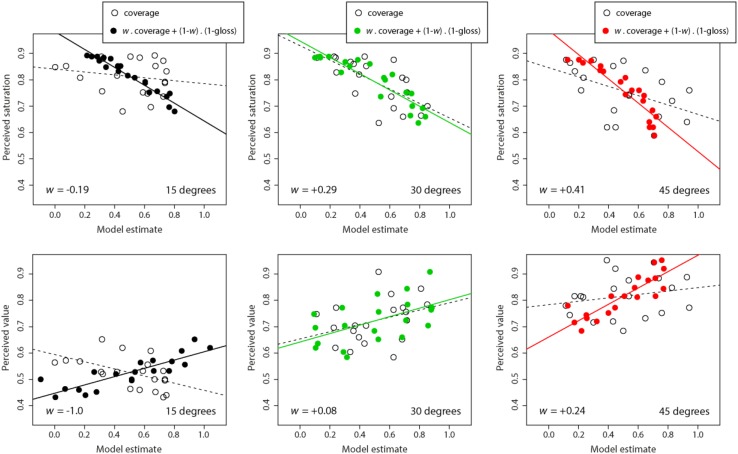
Mean perceived color saturation **(top row)** and perceived color value **(lower row)** plotted as a function of two specular measurements: perceived coverage alone (hollow points and dashed line of best fit) and the linear combination of perceived coverage and inverse of perceived gloss (solid points and line of best fit). Separate axes show data for different surface orientations: 15° **(left)**, 30° **(center)**, and 45° **(right)**. Values for *w* indicate the weight of the linear combination in the model that best predicted variations in perceived color saturation (where −1 ≤ *w* ≤ + 1).

We attempted to model the pattern of data we obtained in perceived saturation and value using coverage alone, as well as a *weighted* linear combination of both coverage and the inverse of perceived gloss estimated in the previous experiment. We used the inverse of perceived gloss because it generated a positive relationship with increasing specular roughness. The weight was allowed to vary between −1 and + 1 to account for situations where coverage may have a negative rather than positive effect on inverse gloss estimates. We anticipated that the emphasis on coverage cues might vary across surface orientations, hence, we parameterized the weight as a free variable when combining coverage and gloss in our model. The results of this modeling are plotted in Figure 12 below and detailed in the next two sections.

##### Modeling perceived saturation

For the 15°condition, the correlation between perceived saturation and coverage alone was not significant (*r* = −0.19, *t*_18_ = 0.83, *p* = 0.42). When we combined coverage with inverse gloss, we found that the best predictor of saturation weighted +1.19 for inverse gloss and −0.19 for coverage. This model correlated very strongly with perceived saturation measured in Experiment 1 (*r* = −0.97, *t*_18_ = 16.55, *p* < 0.00001), accounting for 94% of the variability in perceived color saturation (*R*^2^ = 0.94). This combined model was a better predictor of color saturation than perceived gloss alone measured in the previous experiment (*R*^2^ = 0.65).

For the 30°condition, the correlation between perceived saturation and coverage alone was significant, though moderate (*r* = −0.68, *t*_18_ = 3.91, *p* < 0.005). When we combined coverage with inverse gloss using the weighted linear model, we found that the best predictor of saturation weighted +0.71 for inverse gloss and +0.29 for coverage. This weighted linear model correlated very strongly with perceived saturation measured in Experiment 1 (*r* = −0.90, *t*_18_ = 8.78, *p* < 0.00001), accounting for 81% of the variability in perceived color saturation (*R*^2^ = 0.81). This combined model was a slightly better predictor of color saturation than perceived gloss alone measured in the previous experiment (*R*^2^ = 0.77).

For the 45°condition, the correlation between perceived saturation and coverage alone was significant, though moderate (*r* = −0.48, *t*_18_ = 2.31, *p* < 0.05). When we combined coverage with inverse gloss using the weighted linear model, we found that the best predictor of saturation weighted +0.59 for inverse gloss and +0.41 for coverage. This weighted linear model correlated very strongly with perceived saturation measured in Experiment 1 (*r* = −0.92, *t*_18_ = 9.86, *p* < 0.00001), accounting for 84% of the variability in perceived color saturation (*R*^2^ = 0.84). This combined model was a better predictor of color saturation than perceived gloss alone measured in the previous experiment (*R*^2^ = 0.65).

##### Modeling perceived value

For the 15°condition, there was a significant correlation between perceived value and coverage alone (*r* = −0.52, *t*_18_ = 2.60, *p* < 0.05). When we combined coverage with inverse gloss, we found that the best predictor of value weighted 2.0 for inverse gloss and −1.0 for coverage. This model correlated strongly with perceived value measured in Experiment 1 (*r* = + 0.85, *t*_18_ = 6.87, *p* < 0.00001), accounting for 72% of the variability in perceived color saturation (*R*^2^ = 0.72). This combined model was a better predictor of color value than perceived gloss alone measured in the previous experiment (*R*^2^ = 0.48).

For the 30°condition, there was no significant correlation between perceived value and coverage alone (*r* = + 0.35, *t*_18_ = 1.58, *p* = 0.32). When we combined coverage with inverse gloss, we found that the best predictor of value weighted +0.92 for inverse gloss and +0.08 for coverage. This model correlated moderately with perceived value measured in Experiment 1 (*r* = + 0.54, *t*_18_ = 2.72, *p* < 0.05), accounting for 29% of the variability in perceived color saturation (*R*^2^ = 0.29). This combined model was only a slightly better predictor of color value than perceived gloss alone measured in the previous experiment (*R*^2^ = 0.23).

For the 45°condition, there was no significant correlation between perceived value and coverage alone (*r* = + 0.21, *t*_18_ = 0.93, *p* = 0.37). When we combined coverage with inverse gloss, we found that the best predictor of value weighted +0.76 for inverse gloss and +0.24 for coverage. This model correlated moderately with perceived value measured in Experiment 1 (*r* = + 0.86, *t*_18_ = 7.13, *p* < 0.00001), accounting for 74% of the variability in perceived color saturation (*R*^2^ = 0.74). This combined model was a far better predictor of color value than perceived gloss alone measured in the previous experiment (*R*^2^ = 0.37).

We find that perceived saturation and value were differentially correlated with a weighted linear combination of perceived coverage and inverse gloss. At 15° slant, perceived saturation was negatively weighted toward coverage (−0.19), favoring a greater weighting for inverse gloss. Perceived value was negatively weighted toward coverage (−1.0), favoring a greater weighting for inverse gloss. At 30° slant, perceived saturation was positively weighted toward coverage (+0.29) with proportionally greater emphasis on inverse gloss. Perceived value depended almost exclusively on inverse gloss with little weighting on coverage (+0.08). At 45° slant, perceived saturation was positively weighted toward coverage and the weighting for coverage was yet again higher (+0.41) with slightly higher emphasis on inverse gloss. Perceived value was also positively weighted for coverage (+0.53) with similar emphasis on inverse gloss. These data suggest that the dependence of perceived saturation and value on perceived coverage increases as a function of proximity of the surface’s orientation relative to the primary lighting direction.

## General Discussion

We primarily sought to determine the interdependence of perceived color saturation and lightness on illumination, mesoscopic shape and specular sharpness. To this end, we parametrically varied the orientation of planar surfaces relative to the light source and manipulated mesoscopic relief height and specular roughness. Observers made perceptual color matches to the surface with color described in HSV color space. In Experiment 1, we found significant biases in perception in that perceived saturation declined with increasing specular roughness, while perceived color value increased with increasing specular roughness. The magnitude of these effects was found to be lower when relief heights were greater and lighting was directed along grazing angles relative to the surface (i.e., 15° viewing). This finding supports the view that perceived color depends on the perceptual accuracy in the perceptual separation of diffuse from specular content. In Experiment 2, we also observed interaction between illumination, relief height and specular roughness in the perception of surface gloss. These variations in gloss *per se* only moderately accounted for perceived color attributes. We found that perceived saturation and value could be explained by a computational model that differentially weighted the linear combination of perceptual estimates of gloss and specular coverage (Experiment 3).

The apparent interaction between perceived gloss and lightness could be explained by differences in the perceptual apportionment of specular content attributed correctly to specularity or incorrectly to Lambertian reflectance. Whereas almost all of the specular content is correctly attributed to specular reflectance when specular roughness is low, proportionally more is mis-attributed to Lambertian reflectance when specular roughness is increased (i.e., when their contours are generated by shallow gradients). Previously, Marlow et al. (2012) found that perceived gloss depended on a weighted linear combination of perceived specular sharpness, contrast and coverage. In this study, we examined the usefulness of coverage in accounting for perceived color. The assumption is that color estimates would be better when specular coverage is lower. We found here that perceived coverage was differentially weighted in predicting perceived color saturation and lightness, depending on the orientation of the surface relative to the light source (and the color representation used). Coverage was weighted moderately when surfaces were oriented toward the light source, but inversely weighted when surfaces were more frontally oriented and receiving grazing illumination.

One explanation for the differential dependence of perceived color saturation and lightness on apparent coverage, is that a large amount of specular coverage will contaminate diffuse surface patches used to estimate color. When surfaces with low relief are illuminated with grazing illumination, they generate very few or no specular reflections. This increases the ease at which the diffuse component can be segmented for color attribution. In contradistinction, when surfaces are orientated toward the light source, they generate many specular reflections that increase specular coverage. This increase in specular coverage would contaminate most of the image space where diffuse shading can be used to estimate color attributes. Indeed, reliance on this conflated image structure was found to increase perceived lightness when specular roughness was increased, whilst holding all other reflectance and viewing parameters constant.

The findings of the present study extend the work of previous studies in several ways. Xiao and Brainard (2008) found that perceived color and gloss depended on global illumination and specular roughness. However, they used perfectly spherical objects that did not allow the consideration of megascopic surface orientation *per se*, nor the effect of mesoscopic shape cues on the perception of gloss and color. Schmid and Anderson (2014) showed that perceived lightness depends on mesoscopic shape and specular roughness, but they only considered achromatic viewing conditions. We found that mesoscopic shape affects perceived lightness, even when variations in color saturation are explored. The effects we observe on perceived color were explained in part by variations in perceived gloss and specular coverage. In particular, we found that perceived lightness and HSV saturation were best predicted by not only perceived gloss *per se*, but also the amount of apparent specular coverage across the surface.

These findings together suggest that the dependence of perceived gloss and color on specular sharpness appears to be caused by the perceptual separability of specular from diffuse content. However, there could also be further interactions at a mid-level stage of visual processing that predict perceived gloss and color. For example, Mooney and Anderson (2014) found strong interactions between perceived relief height and specular roughness. Sharp reflections tended to generate percepts of surface curvature that were greater than veridical, compared with surfaces with rougher specular reflections. Variations in perceived lightness have been reported previously across changes in perceived relief height, even when the structure of luminance gradients is preserved (Knill and Kersten, 1991). It is likely that further insight can be gained by examining how perceived shape changes with the effects of relief height and specular roughness we observed in the present study.

When we converted observer color estimates from Experiment 1 from HSV to CIE LCH space, perceptual effects on perceived lightness were preserved, but the previously observed effects on color saturation were diminished on conversion to chroma. Fairchild (2013) defined saturation as perceived colorfulness relative to its own brightness while chroma refers to perceived colorfulness relative to the brightness of a similarly illuminated area that appears white. This means that by using the LCH color space a degree of perceived lightness had already been accounted for and could explain the lack of an effect for specular roughness on perceived chroma. Therefore, we considered a measure of Colorfulness (C^∗^/L^∗^) instead of chroma *per se* to estimate the ratio of perceived chroma to perceived lightness (Schiller and Gegenfurtner, 2016; Schiller et al., 2018). The pattern of main effects we observed in C^∗^/L^∗^ using CIE LCH space was very similar to those we obtained using saturation in HSV color space (Experiment 1). Based on this consistency, we conclude that a similar linear model based on coverage and inverse gloss would account for these perceptual judgments on Colorfulness and Lightness in CIE LCH space.

We propose that the decline in perceived saturation and increase in perceived lightness can be explained by the misattribution of specular highlights to diffuse shading. There are multiple explanations for how this misattribution could be optically determined. Previous work has shown that participants tend to ignore surface regions covered by specular highlights when making judgments of a surface’s body color and lightness (Kim et al., 2012; Toscani et al., 2017). Specular highlights naturally appear near brighter regions of diffuse shading (Koenderink and van Doorn, 1980). Therefore, when surfaces are glossy, participants will tend to estimate saturation and lightness based on darker regions of diffuse shading than they would when surfaces are matte. This is likely given that previous research has shown that participants base their judgments of lightness on brighter diffusely shaded surface regions (Toscani et al., 2013; Toscani and Valsecchi, 2019). However, estimates of color saturation and lightness may have depended more on specular highlight zones when specular roughness was increased in our study. Our light source was white in color and the conflation of specular and diffuse layers would lead to both a desaturation and increase in luminance in image color. Further research using chromatic light sources might offer insight into whether this image-based desaturation and increase in luminance accounts for the perceptual effects we observe.

It may also be worth examining whether motion can help resolve some of the perceived ambiguity in color saturation is motion. Hartung and Kersten (2002) demonstrated that a rotating tea pot could appear to be glossy and uniform in material composition or inhomogeneously textured and matte depending on the pattern of visual motions. A subsequent study proposed the distinction between matte and gloss depends on differences in the velocity field between these materials (Doerschner et al., 2011). Future work could determine whether specular optic flow cues can be used to improve the accuracy of color estimates when specular surfaces are rendered rough.

## Data Availability Statement

The datasets generated for this study are available on request to the corresponding author.

## Ethics Statement

The studies involving human participants were reviewed and approved by the Human Research Ethics Advisory (HREA) Panel, UNSW Sydney (HC14260). The participants provided their written informed consent to participate in this study.

## Author Contributions

All authors listed have made a substantial, direct and intellectual contribution to the work, and approved it for publication.

## Conflict of Interest

QH-T was employed by Canon Information Systems Research Australia (CiSRA) and is now employed with Nearmap. MA and DM were employed by Canon Information Systems Research Australia (CiSRA) and have a financial interest in Bandicoot Imaging Sciences, which is active in the material appearance capture and display industry.

The remaining authors declare that the research was conducted in the absence of any commercial or financial relationships that could be construed as a potential conflict of interest.
